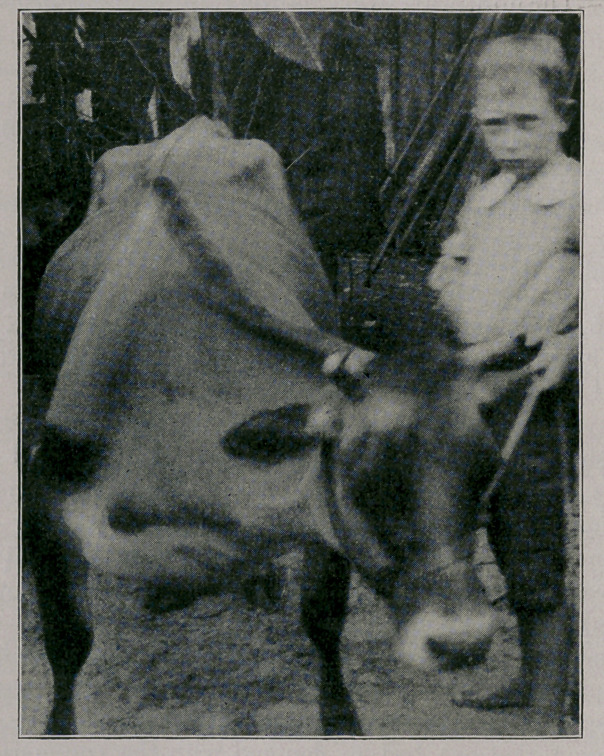# Traumatic Pleuritis and Pericarditis

**Published:** 1901-11

**Authors:** Charles F. Dawson

**Affiliations:** Professor of Veterinary Science, Florida Agricultural College, Lake City, Florida


### TRAUMATIC PLEURITIS AND PERICARDITIS.

By Charles F. Dawson, M.D., D.V.S.,
PROFESSOR OF VETERINARY SCIENCE, FLORIDA AGRICULTURAL COLLEGE, LAKE CITY, FLORIDA.

Recently I was called to attend the Jersey cow "Soda," No. 128,572 of the American Jersey Registry. She was dropped April 15, 1896, and owned by the J. M. Bradner estate, of Tallahassee, Florida, up to April 1, 1901, when she was purchased by Mr. J. C. Bates, of Lake City, Florida.

The symptoms presenting were those of acute indigestion, from which she had suffered for some time. I prescribed the usual remedies. At my next visit I found the animal unimproved, symptoms more pronounced, and expression anxious. Upon a closer physical examination I was able to note a pulsation in the superficial abdominal and jugular veins, all of which were much distended. Gaseous eructations were also occurring at intervals, and these could be induced by deep pressure upon the left flank. The expirations were jerky and accompanied by a grunt. I at once suspected cardiac disease, complicated with pleuritis, and gave the opinion that we had to contend with the traumatic form of heart disease.

At my next visit I noticed œdema, or dropsy of the brisket and neck. It was, at this stage of the disease, impossible to hear the heart sounds or to detect an arterial pulse. Feeling sure of my diagnosis, I advised destroying the animal as a humane procedure. Permission so to do was readily given by the owner, but as the animal seemed easier than at my previous visit, I decided to wait. Subsequent visits revealed a rapid accumulation of liquid in the abdominal and thoracic cavities, and I made arrangements for the post-mortem examination. The next day the animal, in getting to her feet, stumbled, fell, and died. It is presumed the shock of the fall killed her by stopping the weakened heart. The accompanying photograph, showing the attitude of the cow, and the enormously swollen neck and brisket a day or two before death, was taken by my colleague, Professor Hume.

An autopsy was held the following day. The œdematous subcutaneous tissues were infected with putrefactive gas-producing organisms. The chest cavity contained about a gallon of strawcolored fluid, and the abdominal cavity was considerably distended with about ten gallons of similar fluid. The liver was normal, as were also the other abdominal organs, except the spleen, which was entirely devoid of pulp and extremely thin. Upon opening the heart-sac, or pericardium, a pint of purulent, foul-smelling, semi-liquid material escaped. The sac wall was greatly thickened and all adjacent lobes of both lungs were closely adherent to the pericardium and to the chest walls. So much new tissue had been formed around the heart that it was with difficulty the outlines of that organ could be made out. The thoracic viscera were removed as a whole, during which operation careful notice was taken to detect any foreign bodies, such as needles, wires, nails, etc., which might have entered the heart from the stomachs. Although the posterior surfaces of the principal lobes of the lungs had grown to the anterior surface of the diaphragm, as a result of some irritant, no foreign bodies were noticed up to this time. The heart was cut away from the adherent lungs and carefully dissected the next day in the hope of locating the foreign body, which I now felt sure was present. Very little work revealed the cause of the whole trouble, for lying loose in the heart-sac, or pericardial cavity, was the sharp end of a hat-pin, two inches in length. This had evidently been swallowed by the animal, and on account of its sharp point had entered the wall of the stomach, worked its way through that organ, through the diaphragm, and into the sac around the heart, where it started up an intense inflammatory process, which resulted in the formation in that serous cavity of an abundant exudation. This became infected with the bacteria adhering to the pin, and finally formed the ill-smelling pus discovered. The heart itself was very small, flabby, and roughened on its surfaces from inflammatory deposits, the great atrophy or wasting and the large amount of pus accounting for the absence of the arterial pulse and heart sounds during life. The disease was, therefore, notwithstanding its severity, a chronic one. If this case teaches anything, it is this : that it is extremely bad practice to allow cattle or any ruminant to feed near a human habitation ; that sweepings from the dwelling should not be thrown upon pastures ; that clippings from wires, nails, and other metals used in the construction of lines of telephones and telegraph, buildings, implements, fences, and baling-wire should not be allowed where cattle graze. It has been said that it does not pay to pick up nails. This may be true in some cases, but it is certainly not true in regard to pastures. Even a pin is capable of causing this disease, and, in fact, is a much more dangerous object than a blunt six-inch spike could be, not only because it would not likely be discovered, but because it is sharp-pointed, it being only sharp-pointed objects that can penetrate the stomach walls and pass into the heart or other organs. It is a common occurrence to find nails, old shoes, combs, hair-brushes, pieces of wood, etc., in the stomachs of cattle. These may or may not cause trouble—they are most certainly not beneficial—but they are not capable of causing the disease under discussion, because they are not sharp-pointed.

The maw cleaners at the stock-yards in Chicago save, as curios, all the foreign bodies found in their work. I once saw an exhibit of them, and noted the following articles which had been removed from the stomachs of cattle slaughtered for beef at Chicago: A jack-knife, watch-chain, silver dollar, hair-pins, large nails, a bridge-bolt 11¾ inches long by ¾ inch thick, with a square head and carrying a nut; the most curious of all being a sulky rake-tooth measuring four feet in length, and curved as when in use. This enumeration is made only to show that cattle are extremely careless of the way in which they feed, and that they can carry without apparent inconvenience an absurd collection of foreign bodies in the stomach, provided they are not sharp-pointed. In fact, the first feeding is simply a gathering of food and passing it into the large first stomach, paunch, or rumen. It is a sort of store-room for food, which here undergoes a certain amount of softening and starch fermentation preparatory to its being belched up later as "cud," and given a thorough mastication and insalivation. Little is the wonder that cattle swallow all sorts of indigestible bodies, some of which are capable of doing great harm or even causing death. Such occurrences are not, then, due to a depraved appetite in most cases. In the case of long objects, such as the rake-tooth, it was voluntarily swallowed by the animal to a certain distance, but after that point in the throat was passed, swallowing was no longer a voluntary act, but the muscles of the throat grasped the object, and it was carried on into the stomach reflexly, or involuntarily. One can prove this on himself at any time by attempting to return something to the mouth which has once passed the back of the tongue.

Foreign bodies do not always enter the heart, but may be found in the lungs, where they cause pleurisy and pneumonia; or they may be found in the liver, where they produce abscesses which may or may not end in death. Cases are recorded where foreign bodies have traversed the body, making their exit at distant points. This occurs sometimes in people, especially in the insane, some of whom are prone to swallow indigestible substances.

Although traumatic heart disease is not frequently reported, there can be little doubt that at least in those cases of sudden and mysterious deaths of cattle occurring in, around, or near human habitations a large percentage of them are due to injuries (traumatisms) from swallowing sharp-pointed, indigestible substances. There is no treatment for the condition, but it can be largely prevented, which is better. It is little or no trouble to pick up nails, wires, and other metallic bodies when seen either on the lawn or pasture to which the cattle have access, or from the hay fed them, and thus save the life of a valuable animal.

As regards the kinds of pastures upon which the most dangerous objects are found, it may be stated that those nearest girls' dormitories or recreation grounds easily take the lead as producers of this trouble. Of thirteen head of cattle belonging to a girls' school, and killed by a government official because of their being tuberculous, all had disease due to swallowing foreign bodies. In another herd of twenty-eight cattle belonging to a boys' school, and destroyed for the same reason, only one had disease which was traceable to the presence of a foreign body.

## Figures and Tables

**Figure f1:**